# Proposition of a transdiagnostic processual approach of emotion dysregulation based on core triggers and interpersonal styles

**DOI:** 10.3389/fpsyt.2024.1260138

**Published:** 2024-02-07

**Authors:** Martin Blay, Miguel Duarte, Marie-Alix Dessouli, Amaury Durpoix, Eva Rüfenacht, Sébastien Weibel, Mario Speranza, Nader Perroud

**Affiliations:** ^1^ ADDIPSY, Addictology and Psychiatry Outpatient Center, Santé Basque Développement Group, Lyon, France; ^2^ Centre de recherche en Epidemiologie et Sante des Populations Team ‘DevPsy’, INSERM, Universite Paris-Saclay, UVSQ, Villejuif, France; ^3^ Psychiatric Specialties Unit, University Hospitals of Geneva, Geneva, Switzerland; ^4^ Department of Emergency Psychiatry, University Hospital Edouard Herriot, Hospices Civils de Lyon, Lyon, France; ^5^ Department of Psychiatry, University Hospitals of Strasbourg, Strasbourg, France; ^6^ U1114, INSERM, Strasbourg, France; ^7^ University Department of Child and Adolescent Psychiatry, Versailles Hospital Center, Le Chesnay-Rocquencourt, France; ^8^ Department of Psychiatry, University of Geneva, Geneva, Switzerland

**Keywords:** emotion dysregulation, trigger-based approach, processual, transdiagnostic, borderline personality disorder

## Abstract

Emotion dysregulation (ED) has primarily been described in patients suffering from borderline personality disorder (BPD) and is an integral part of this diagnosis, but it is also a transdiagnostic construct that can be found in several other psychiatric disorders. The strong relationships between ED and BPD may lead clinicians to underestimate ED associated to other clinical contexts. This can lead to difficulties in diagnostic and treatment orientation, especially in the context of comorbidities. In this article, after reviewing the literature on the development and functioning of emotion dysregulation, and on the evidence for emotion dysregulation in eight disorders (borderline personality disorder, pathological narcissism with/without narcissistic personality disorder, obsessive-compulsive personality disorder, antisocial personality disorder, bipolar disorder, autism spectrum disorder, complex post-traumatic stress disorder, and adult attention deficit hyperactivity disorder), we present a transdiagnostic processual model of emotion dysregulation based on core triggers and interpersonal styles to try to address this issue and to provide a simple but technical tool to help clinicians in their diagnostic assessment and treatment orientation. By focusing more on typical patterns and interpersonal dynamics than only on categories, we believe that this model may contribute to the actual need for improvement of our current psychiatric classifications, alongside other well-studied and under-used dimensional models of psychopathology (e.g., HiTOP, AMPD), and may be useful to build more specific treatment frameworks for patients suffering from ED.

## 1 Introduction

Emotions can be defined as whole-body phenomena, that include changes in subjective experience, behaviors, and physiology, and that emerges in response to a specific object/situation perceived as relevant for oneself (1). On the other hand, emotion regulation can be defined as the way people attempt to influence this phenomenon, using different type of strategies (2). One of the most popular model of emotion regulation development and difficulties is the biosocial model developed by Marsha Linehan in patients suffering from borderline personality disorder (3). According to the latter, emotion dysregulation (ED) arises from the encounter of biological vulnerabilities (that include a lower threshold for emotion triggering, a larger increase in emotion intensity, and a slower decrease afterwards) with an *invalidating environment*, which can be defined as an environment that persistently ignores, disregards or punishes emotion expression. Invalidation ranges from pervasive minimizing and criticizing of emotional expression to physical and sexual abuse. Progressively in their development, these patients face more and more emotional crises, and start undergoing dysfunctional behaviors to regulate their emotions (including non-suicidal self-injury (NSSI), impulsive or suicidal behaviors or interpersonal conflicts), which may lead to the maintenance and/or aggravation of ED. This pioneer theoretical work has allowed the development of a manualized and validated therapy for BPD patients, Dialectical Behavioral Therapy (DBT), which has been shown to be effective notably in reducing self-harm behaviors or suicide attempts (4, 5). Finally, apart from DBT, other manualized treatments aiming to treat emotion regulation issues have been developed. These treatments, even though also focusing on emotion regulation, are often based on related but different conceptualizations of ED. The most studied remains mentalization-based therapy (MBT), another treatment initially developed for BPD patients, postulating that emotion dysregulation is linked to impairments in the development of mentalizing abilities (i.e., the ability to understand and process the combination of emotions, beliefs, intentions, thoughts and motivations that underlies our own and others’ behaviors) (6).

As mentioned earlier, ED has primarily been described in patients suffering from BPD, a disorder marked by important difficulties in emotion regulation (7). ED is an integral part of the borderline diagnosis, as emphasized by the presence of the construct and of its related symptoms (NSSI, recurrent suicide attempts, impulsive behaviors, interpersonal conflicts…) in the list of the 9 criteria. In this context, clinicians often associate these symptoms with the BPD diagnosis, and patients with ED often fulfill criteria for BPD, even if it is now well-known that ED can also be encountered in many other psychiatric disorders than BPD, and is currently considered as a transdiagnostic construct (8–10). In the field of personality pathology, patients suffering from pathological narcissism (PN) (with/without narcissistic personality disorder, NPD), obsessive-compulsive personality disorder (OCPD) and antisocial personality disorder (ASPD) may also present several of the above mentioned symptoms of ED (11–18). Apart from the field of personality disorders, ED can also be found in patients suffering from bipolar disorder [BD, (19, 20)], autism spectrum disorder [ASD, (21, 22)], complex post-traumatic stress disorder [cPTSD, (23)] and adult attention deficit hyperactivity disorder [ADHD, (24, 25)]. These disorders may therefore be diagnosed as BPD at the behavioral level, even though they don’t share BPD specific psychopathological issues. This could be linked with the high BPD comorbidity rates found in these disorders. Indeed, studies have reported a 37% prevalence rate of BPD in NPD patients, of 16.6% in OCPD patients, and of 21% in ASPD patients (26). Regarding bipolar disorder, empirical studies have found a 20% prevalence rate of BPD in patients with type II bipolar disorder (27). Regarding trauma, 25–30% of adults meeting criteria for either PTSD or BPD also meet criteria for the other disorder, and BPD is comorbid in 8-79% of cPTSD cases (23). Regarding neurodevelopmental disorders, prevalence rate of BPD is 27.2% in patients with adult ADHD (28) and between 0 and 10.6% in patients with ASD (29). Given these issues, when facing patients suffering from ED, the distinction between these disorders, BPD, and true comorbid presentations remains a common clinical challenge.

If categories are useful [because they offer important opportunities in terms of psychoeducation and treatment orientation, and because they provide a global meaning to suffering that patients can often easily understand (30)], the categorical approach has been the subject of large criticisms over the last decades. Indeed, describing mental health issues in taxa and categories (such as the DSM-5 does) is often considered as not supported by empirical data, the latter indicating that these issues should be considered dimensionally (31). These considerations have led researchers to develop new dimensional ways of conceptualizing psychopathology. One of the most developed dimensional conceptualization is the Hierarchical Taxonomy Of Psychopathology (HiTOP) model (32, 33). According to this model, psychopathology should be understood as a hierarchy of spectra. At the top of the model lies the super-spectrum dimensions, including the well-known general factor of psychopathology “p”, extensively studied and described by pioneer researchers like Caspi (34) and considered as underlying every dimension of psychopathology. These general dimensions can then be divided in several spectra (e.g., internalization, thought disorders, disinhibited externalization, antagonist externalization, detachment, somatoform), many being subdivided in several subfactors (e.g., the internalizing spectra can be divided into sexual and eating disorders, fear, distress and mania) (32). In order to cross these new conceptualizations with the most commonly used conceptualization of mental disorders (categories) and to make it more readable for non-specialists, the HiTOP model also indicates in which dimensions each of the well-known categories can be found, one category being not restricted to one dimension (e.g., the BPD category can be found both in the distress subfactor of the internalizing spectra and in the antagonist externalization spectra, which is representative of the large spectrum of expression this disorder may have). To note, the spectra constituting the model are still evolving, with several super-spectra being still described and proposed [e.g., (35, 36)]. Regarding the main subject of this paper (emotion dysregulation), some authors have described a possible “emotion dysfunction” super-spectrum, composed of the internalizing and somatoform spectra (36), emphasizing the transdiagnostic aspect of emotion regulation issues. Moreover, this movement of dimensionalization of mental health disorders also has its correlates in specific fields, for example in the personality disorder field with the development of dimensional models like the Alternative Model of Personality Disorders of the DSM-5 (7) or the ICD-11 model of personality disorders (37), both describing personality pathology as the association of both a general factor of personality functioning (criterium A) and up to 5 specific domains of personality traits (criterium B). However, these new models, although highly inspiring to overcome classic categorical approach, are currently hardly implemented in common clinical practice (32, 38), where categorical approach remains the most used, even though work groups are working on implementing dimensional approaches (32, 39). Finally, these models are also not free of criticisms, either on their overall utility [e.g., for HiTOP (40)] or on their representativeness of the clinical complexity of patients [e.g., for AMPD/ICD-11 (41)].

The fact that the main commonly used approach in clinical practice remains the categorical one may be particularly problematic in the context of ED. Indeed, these disorders have specific core psychopathological and interpersonal issues, and a confusion between them (or on which to prioritize in case of comorbidity) may have an impact on treatment orientation and outcomes. For example, BPD criteria (and thus emotion dysregulation symptoms) is thought to represent a general core feature of personality pathology severity and may be found in severe personality disorders more as an indicator of the level of personality functioning than as an indicator of underlying *borderline* processes (42). Thus, by focusing on a pure categorical approach, severe OCPD and NPD patients may be also diagnosed with BPD and may thus be seen as *classic* borderline patients, which may lead clinicians to miss core underlying issues and impact the course of treatment [given that, for example, pathological narcissism is associated with drop out in psychotherapy (43)]. Following the same rationale, when facing ASD patients with ED symptoms, clinicians may focus only on externalized symptoms (like NSSI or anger outbursts) and under-estimate typical autistic traits [e.g., cognitive rigidity, hypersensoriality), even though it seems important to take them into account in the treatment process (for example, some authors have suggested the importance of a quiet and stable environment in DBT programs for ASD patients (44)]. Moreover, when considering PTSD/cPTSD, an overfocus on associated ED-symptoms may also have consequences on treatment, given that – for example - several authors suggested that complex PTSD should be treated first when comorbid with BPD given that these patients are often too vigilant to be challenged (45). Finally, this could also have consequences in patients suffering from ADHD or BD, given the importance of medication in these disorders and the global consensus on its unusefulness in BPD (46).

In this context, we believe that a simple, transdiagnostic processual approach based on core triggers of emotion dysregulation (i.e., what s*pecifically* induces it in each disorder) could be useful for clinicians and could provide a complementary perspective to the classic categorical approach. We also believe that the investigation of recurrent and typical interpersonal issues could enhance such approach. Therefore, the aim of this paper is to describe this approach, after reviewing the literature on the development and functioning of emotion dysregulation, and on the presence and characteristics of ED in eight disorders (with a focus on the main relevant triggers and interpersonal styles): borderline personality disorder, pathological narcissism with/without narcissistic personality disorder, obsessive-compulsive personality disorder, antisocial personality disorder, bipolar disorder, autism spectrum disorder, classic and complex post-traumatic stress disorder, and adult attention deficit hyperactivity disorder. To note, this list is non exhaustive, as other disorders are linked to ED (e.g., substance use disorder). Our aim is to provide a simple and easy to implement transdiagnostic model of emotion dysregulation to help clinicians in their daily practice, in terms of diagnostic assessment, but also in terms of psychotherapeutic treatment orientation.

## 2 How does emotion (dys)regulation work and develop?

In the introduction, we described Marsha Linehan’s model of development and expression of emotion dysregulation. Even though this model has gained large audience over the last decades, notably linked to the efficacy of its associated psychotherapy to treat BPD, many other models, often more complex, have been built (and are currently improved) to better understand how emotion dysregulation works and how it develops in one individual (47). In this paragraph, we want to provide a larger picture of the literature regarding on how emotion regulation works and develops, and to present why we believe our trigger-based approach may be relevant with regards to these conceptualizations.

### 2.1 Emotion regulation development and processes

Emotions are adaptive. They are necessary for survival, allowing the subject to evaluate the significance of his/her environment for well-being, to organize behaviors to achieve and maintain this well-being, and to communicate about these goals (48). Emotions are generated when facing a situation considered relevant for oneself, leading to attentional focus on the important aspects of this situation, appraisal of these aspects in relation to one’s aims and goals, and finally to physiological and behavioral response (2). Thus, emotions should not be restricted to the physiological and behavioral response; and, as a corollary, emotion regulation (ER) should not be considered only as a rigid ability to regulate this response (49). Instead, ER can be better defined as a dynamic set of extrinsic and intrinsic processes responsible for monitoring, evaluating and changing the intensity and temporality of emotional reactions, that one’s uses to achieve his/her goals, taking into account social expectations (50). Such definition is more helpful and accurate as it encompasses the other domains of emotion generation and considers the context in which the emotion arises and the goals underlying emotion regulation. Several theoretical models have been developed to describe the different ways subjects engage in ER and determine which strategies are the most effective and adaptive (2). One of the most used is the process model of emotion regulation develop by Gross and colleagues (51). According to the latter, the ER cycle starts when there is a discrepancy between *desired* and *actual* emotional state. When facing such discrepancy, the subject 1°) identify the opportunity for regulation, and 2°) select and 3°) implement one or several of the 5 main emotion regulation strategies (namely, situation selection and modification, attentional deployment, cognitive change, and response modulation), the whole cycle being monitored for the efficacy of emotion regulation (2). This model, among other, allows clinicians to consider emotion regulation more holistically than models focusing only on emotional arousal.

But, if these dynamic processes are central for the subject’s adaptation to his/her environment, how do they develop in one individual? Even though this question is not fully answered and is still being studied nowadays (47), development and acquisition of emotion regulation during development is mostly considered as a complex and continuous phenomenon (52, 53). At birth and during the first months, toddlers, when activated, are only regulated by their environment (54). Then, progressively, the baby/child translate from being regulated by others (*external regulation)* to being self-regulated (*internal regulation*). This is allowed notably through progressive development of intra psychic strategies (i.e., attentional redirection, cognitive reevaluation), flexibility (i.e., ability to adapt the strategies to one context, possible use of multiple strategies), specification (i.e., ability to use specific strategies for each emotion/situation), and integration of social norms and goals (55). However, this development also relies on the type of environment in which the subject grows, and notably on the interaction with caregivers. Indeed, emotion regulation acquisition must also be considered under the light of attachment theory (56). Attachment can be defined as a universal and specific human need of security in relationships, notably between the new-born baby and his/her caregivers (56). Indeed, toddlers have the innate ability to seek closeness to the caregiver through skills allowing the mobilization of the caregiver’s interest and attention (e.g., crying, clinging, etc.). Normally, the caregiver is attracted and can therefore intervene to try to help the baby regulate his or her experience. Such regulation ability acquisition develops notably through contingent, congruent, and marked parental mirroring in early interactions. If these interactions are adequate, they will allow the child to feel safe enough to explore and learn from the environment around him/her, an aspect that is crucial for the development of emotion regulation abilities, notably because it confers the ability to learn how to select relevant information in the environment. On the other hand, when these interactions are inadequate, the child will experience low reliability and availability of the attachment figure, which impedes the possibility to securely explore the environment and to develop productive emotion regulation abilities (57, 58).

Altogether, emotions cannot be defined only as physiological and behavioral manifestations. They arise in specific, attention-grabbing, relevant situations, and their regulation is a complex process including identification of the opportunity to regulate, selection of the most relevant strategies, and implementation of these strategies. These abilities are built continuously during development, where subjects go from external regulation to internal regulation, this process requiring safe and adequate interactions with caregivers. But how can these processes go awry?

### 2.2 Development of emotion dysregulation

Emotion regulation is thus a core aspect of human experience and interactions, notably to attain and maintain well-being. However, this dimension can become problematic when an individual’s emotion regulation patterns impair developmental goals in short and/or long-term time frame (47). According to specialized literature, impairments are considered either when interfering with achieving short and/or longer term goals for well-being, when violating developmental expectations for appropriate behavior, or when violating sociocultural standards for emotion-related communication and behavior (47). Thus, emotion dysregulation is not necessarily characterized by having negative, overwhelming, or sustained emotions. Instead, the level of impairment depends on the context in which emotions arise and on how the subject deals between the well-being goals and the reality of the environment (47, 59). Indeed, some patterns can be adaptive *in the immediate context* but can become maladaptive at a larger time frame. For example, in the case a child whose parents punishes (e.g., through physical abuse) their offspring’s sadness expression, some pattern of emotion regulation (e.g., emotion suppression) can be efficient for short term goals (i.e., self-protection). However, these same patterns, learnt and integrated in a specific environment, may also become irrelevant when the child grows up and when the context changes (e.g., when the offspring leaves his/her parents’ house and start to build his/her adult life). Such emotion regulation patterns may therefore lead to impairments in long-term goals for well-being (e.g., inability to develop close relationships due to avoidance of situations requiring vulnerability expression). In this example, it is more the discrepancy between *past* emotion regulation patterns and *actual* context (e.g., less abusive and more secure social interactions) that lead to emotion dysregulation, rather than the strategy used or the intensity of emotions. Finally, the same observation can be made in terms of attachment. When early interactions and parental mirroring are inadequate, insecure attachment styles may develop. These attachment patterns (except for disorganized attachment) can be considered as adaptive reactions to real-life experiences, because they allow the subject to survive in an environment that does not fulfill his/her needs (56). However, this perceived absence of reliability and security of environment, when integrated, can become problematic when the context changes and when relational threats become less common.

Altogether, emotion dysregulation cannot be limited to the valence or intensity of the emotion arousal nor to a deficit in the use of emotion regulation strategies. Instead, emotion dysregulation can be better understood as a mismatch between 1°) specific emotion regulation patterns developed to fulfill well-being goals in a specific environment and 2°) the reality of the *actual* environment, that need different emotion regulation patterns to fulfill short and long-term well-being goals.

### 2.3 Specificities in terms of triggers of emotion dysregulation

It is important to note that the considerations we presented in this section are general and not restricted to clinical population. However, emotion dysregulation is a transdiagnostic risk factor for psychopathology (60, 61) and it can be found in a large number of psychiatric disorders (8). At this point, readers may ask how these processes (i.e., process model of emotion regulation, context-dependent conceptualization of emotion dysregulation…) can be differentially impaired in a way that leads to different categories of psychopathology. Such important question may require large theoretical and empirical works to be fully answered. Here, we will only focus on the aforementioned conceptualizations, and describe how, in our perspective, the triggers we present below can be understood under this lens.

First, emotion dysregulation may develop and express differentially depending on the actual biological and neurocognitive background associated with the disorder. This may be particularly relevant (but not exclusively) for neurodevelopmental and bipolar disorders. Indeed, depending on underlying neurocognitive functioning, the identification of relevant situations and/or the access to the five main emotion regulation strategies could be impaired. For example in ASD patients, as suggested elsewhere (22, 62), impaired ability to filter environmental stimuli may impact the ability to adequately identify and select relevant situations, and intolerance to change and cognitive rigidity may be associated with a lower ability to use cognitive change strategies. These neurocognitive specificities, and their associated emotion regulation issues, may lead to specific hypersensitivities/triggers in this population. The same rationale may also be applied for ADHD and bipolar disorders, where disorders’ associated executive function deficits also impact emotion regulation processes (63, 64). Altogether, if the classic processes at play in emotion regulation can be impaired by neurocognitive deficits, and if different disorders have different types of neurocognitive alterations, it seems plausible to assume that each disorder may be associated with specific patterns of emotion regulation dysfunction and specific hypersensitivities/triggers, depending on the underlying neurocognitive impairments. This aspect could be the first level of analysis when considering disorders’ specificities in emotion dysregulation.

Second, emotion dysregulation may develop differentially depending on the context in which emotion regulation abilities were developed. This may be particularly relevant (but not exclusively) for personality disorders or cPTSD. Indeed, depending on the type of environment the patient grew up, he/she may be confronted to different types of interpersonal threats, attachment issues, and may develop different types of emotional strategies to attain immediate well-being. All these aspects may foster different types of sensibilities. For example, a patient growing up in a familial context punishing failures, weaknesses, and communicating either directly or indirectly that one cannot be loved if failing, may be at a higher risk for specific sensibilities related to self-esteem. Thus, situations that may not be considered as relevant in terms of self-esteem by most people (e.g., not being congratulated for work) may be identified as so by the subject and may trigger emotion generation. Moreover, in such invalidating context, the patient may be at higher odds of developing emotion regulation strategies like emotional suppression (to not communicate vulnerabilities) and avoidance of difficult situations (to avoid failure). These patterns of emotion regulation could be thus considered adaptive in such environment, to attain immediate well-being (e.g., less punishment and critics, more closeness to caregivers). However, these same patterns (e.g., never showing vulnerabilities, avoiding difficulty) may also become maladaptive in a less adverse environment (e.g., in a social sphere where defaults are not punished or criticized). Altogether, such patient, by experiencing emotion generation more often when facing self-esteem threats, and by maintaining the use of formerly adaptive emotion regulation patterns, may be impaired (e.g., having repeated conflicts in workplace, frequent job quitting) in his/her ability to fulfill long-term well-being goals (e.g., building a career), which could lead to the diagnosis of pathological narcissism. In resume, the environment development and the context in which emotions arise may influence how emotion regulation develops and expresses, which may once again lead to different ways of emotion regulation dysfunction, and to different hypersensitivities/triggers. This aspect could be the second level of analysis when considering disorders’ specificities in emotion dysregulation.

Even though our review may be seen as oversimplifying and partial, we believe that these two dimensions (the influence of underlying neurocognitive impairments and of the type of environment in which emotion regulation abilities developed) could – at least partly- explain the different in terms of triggers of emotion dysregulation between the 8 disorders we will present in the paragraph 3. Moreover, given that these triggers could be seen as the expression of different impairments in the emotion regulation cycle, the *trigger-based* psychotherapeutic approach we present in paragraph 5 may also be relevant to target these specific impairments indirectly but more specifically, compared to general approaches focusing on the whole cycle that may miss each disorder specificities.

## 3 Emotion dysregulation, triggers and interpersonal style

In this paragraph, we will review the evidence regarding emotion dysregulation in the aforementioned disorders, with a focus on specific triggers and interpersonal styles. A summary can be found in **Table 1**.

**Table 1:** Summary of specific triggers and interpersonal styles.

Disorder		Trigger	Interpersonal style
*Personality disorders*	**Borderline personality disorder**	Real or imagined rejection and abandonment	Intense and unstable patterns of idealization and devaluation, emotional and identity dependency toward others
	**Narcissistic personality disorder/Pathological narcissism**	Real or imagined self-esteem threats	Dependency on external admiration, validation or reassurance to regulate self-esteem, arrogancy and devaluation towards others, victimization
	**Obsessive-compulsive personality disorder**	Internal or external threat to perfection, order, or control	Tendency to overcontrol others’ behaviors, or to avoid relationships because of fear of not meeting other’s expectations. Relegation of relationships after one’s productivity and effort.
	**Antisocial personality disorder**	Threats to power and dominance over others	Tendency to manipulate, lie, and exhibit aggressiveness toward others. Relations are marked by dominance and intimidation, with a lack of concern and remorse. Others are seen as ways to reach personal gains.
*Other disorders*	**Bipolar disorder**	No specific triggers for ED (autonomous variations), but not for mood episodes.	Euthymic bipolar patients have more stable relationships than BPD patients, but may present alterations linked with impulsivity, persistent depressive symptoms and neurocognitive impairments.
	**Complex post-traumatic stress disorder**	Reminders of traumatic events and trauma-impacted beliefs about self and relationships	Severe emotional detachment, tendency to avoid relationships, mistrust, feeling of worthlessness
	**Autism spectrum disorder**	Difficulties in filtering environmental stimuli (including sensory), cognitive rigidity (notably intolerance to change)	Lack of understanding of social norms, lack of willingness to enter in relations, lack of understanding non-verbal communication and social reciprocity
	**Attention deficit hyperactive disorder**	Impatience, boredom	Too talkative and excitable, impulsivity and novelty seeking leading to logistical and organizational issues

### 3.1 Personality disorders

#### 3.1.1 Borderline personality disorder

Borderline personality disorder (BPD) is a common and potentially severe personality disorder that affects around 1.6% of the general population (65). It is characterized by difficulties in emotion regulation, with an unstable sense of identity and intense and conflictual interpersonal relationships, associated with impulsive behaviors, self-harm, and repeated suicide attempts (7). Even though different conceptualizations of the disorder have been described over the last decades (3, 6, 45, 66, 67), several authors have emphasized that the core trigger of BPD symptoms is fear of rejection and abandonment (45, 68). When considering interpersonal style, the specificity of BPD patients lies in hyperintense and unstable relationships, with fluctuations between idealization and devaluation of others and concomitant fluctuations in emotions and sense of identity, dimensions that are mainly associated with the perceived quality of the interpersonal relationships. In this context, patients suffering from BPD can often threaten or beg others to not abandon them, with sometimes the use of self-harm or suicidal threats to avoid abandonment (45, 65).

#### 3.1.2 Narcissistic personality disorder/pathological narcissism

Narcissism is a normal and important psychological function that describes one’s capacity to regulate self-esteem without relying on social environment validation or self-enhancement. Development of normal narcissism is a continuous phenomenon that may be altered, thus resulting in the development of pathological narcissism, that may be defined as significant deficits in self-esteem regulation and use of maladaptive strategies to cope with self-esteem threats (69). Two non-exclusive forms of pathological narcissism (depending on the patient’s reaction when facing self-esteem threats) have been described*. Grandiose* narcissism is characterized by a tendency to use self-enhancement, entitlement, fantasies of power and fame and exploitative or aggressive behaviors, whereas *vulnerable* narcissism is more characterized by internalized feelings of shame, depression, with a tendency to self-criticism, social withdrawal, and a heightened risk of suicidal attempts (69, 70). From a categorical perspective, the related disorder of the DSM is narcissistic personality disorder (NPD), with a prevalence in general population up to 1% (71). Many criticisms has emerged regarding the clinical validity of the NPD diagnosis, given its overemphasis on narcissistic grandiosity and the lack of consideration of narcissistic vulnerability, which may explain its low prevalence rate compared to the prevalence of pathological narcissism as a construct in clinical practice (69, 72). Narcissistic patients also struggle with emotion dysregulation (13, 17, 73), especially with emotion like anger/rage, shame and envy (74), and show several ED-related behaviors [e.g., NSSI (12)]. According to current research, the main trigger of emotion dysregulation in narcissistic patients is real or perceived self-esteem threats (75). Moreover, regarding interpersonal style, as emphasized by the alternative model of the DSM-5 (7), the specificity of narcissistic patients lies in the place given to relationships, that often exist mainly to regulate self-esteem, with excessive reference to others for self-definition, goal setting based on gaining approval from others, over-sensitivity to others’ reactions (but only if they are perceived as relevant to oneself), and with a tendency towards arrogancy, devaluation of others and victimization when feeling threatened.

#### 3.1.3 Obsessive-compulsive personality disorder

OCPD is the most frequent personality disorder that affects around 4.3% of the general population (76). It is associated with a significant number of psychiatric comorbidities, including anxiety and mood disorders, even though OCPD patients have a better clinical functioning than patients with BPD (77, 78). According to several experts, OCPD is a disorder characterized by difficulties in tolerating loss of control, imperfection or disorderliness (79), and OCPD patients can be separated in two subtypes: *anxious* type (reacting to control threats with self-criticism, anxiety, worrying, procrastination and social avoidance) and *controlling* type (reacting to control threats with detachment, irritability, criticisms and judgment, or anger) (80). Moreover, patients with OCPD also struggle with high perfectionism, with a sense of self-worth overdependent on striving and achievement that leads to counterproductive behaviors and self-criticism when these patients fail to meet the inflexible standards they place on themselves (81). Thus, when facing internal or external threats to control or meeting their high standards, these patients (mostly – but not exclusively – the *controlling* type) may experience emotion dysregulation and related symptoms, like interpersonal issues or self-harm (11, 18, 82). Regarding interpersonal style, OCPD specificity lies in the view of relationships as secondary to work and productivity, with rigidity and stubbornness combined with high moral standards for others when these patients enter in relation (controlling type), or with a tendency to be submissive, self-critical and over-attentive to not meeting expectations of others (anxious type) (7, 79).

#### 3.1.4 Antisocial personality disorder

ASPD is one of the most common personality disorder in the general population, with a prevalence rate around 3% (76), and is described in the DSM-5 as a « pervasive pattern of disregard for and violation of the rights of others », with clinical features such as failure to conform to social norms with aggressiveness, deceitfulness and lack of remorse (7). It is associated with increased morbidity (both psychiatric and somatic) and mortality (with – for example – 4 to 5% of ASPD patient completing suicide) (83). It is also associated with extended harm and large societal costs, notably due to aspects like interpersonal aggressiveness & criminality (84). ASPD patients are also well-known to be prone to emotion dysregulation and related aspects like NSSI or impulsive behaviors (15, 16). Regarding triggers, ASPD seems to be specifically triggered by threats to power and dominance over others (e.g., by hierarchy, societal norms or barriers to obtain personal gains), which can be linked to fear of exploitation and victimization by others and to fundamental beliefs such as « I should hurt him before he hurts me » (85). Regarding interpersonal styles, as emphasized by the AMPD, the specificity of ASPD patients lies in a tendency to manipulate, lie to and sometimes aggress others, in order to maintain dominance and intimidation, with a clear lack of concern and remorse about negative effects on others, that are often seen as ways to fulfill personal needs (7).

### 3.2 Other disorders

#### 3.2.1 Bipolar disorder

Bipolar disorder (BD) is a common and severe psychiatric disorder that can be defined as the presence of manic episodes (type I), or the concomitant presence of hypomanic and depressive episodes (type II) (7). It has an overall lifetime prevalence of 0.6% for bipolar type I and 0.4% for bipolar type II (86), and it is associated with significant impairment in professional, social, and familial functioning (87). BD patients also often struggle with emotion dysregulation (19), and engage in ED-related behaviors like NSSI (88), impulsive and suicidal behaviors (89). Notably, emotion dysregulation can be found in euthymic bipolar patients (90). Specific triggers are often found in mood episodes (91, 92), with some authors suggesting different types of triggers for depressive and manic episodes. However, regarding emotion dysregulation and affective instability, several authors have proposed that ED in BD patients may not have specific triggers compared to patients with BPD, with emotional swing being quite autonomous and non-driven by specific types of interpersonal or intrapsychic events (27). Moreover, regarding interpersonal style, euthymic bipolar patients are thought to be able to maintain relatively stable relationships compared to BPD patients (93), with a lower tendency to avoid relationships due to fear of rejection (27, 94). However, interpersonal functioning can also be altered in euthymic bipolar patients, notably linked to impulsivity, persistent depressive symptoms, and persistent neurocognitive impairment (95, 96).

#### 3.2.2 Complex post-traumatic stress disorder

Post-traumatic stress-disorder (PTSD) is a common mental disorder affecting around 5.6% of the general population (97) and is defined in the 11^th^ version of the ICD (37) as the occurrence of three symptoms clusters after the onset of a traumatic event: re-experiencing, avoidance of reminders, and a perception of heightened current threat (98). However, observation of chronically traumatized patients that experienced repeated physical or sexual abuses have underlined that these patients often present more complex presentations than classic PTSD patients due to an effect of prolonged traumatisms on self-organization (99). To address this issue, a new diagnosis of complex post-traumatic stress disorder (cPTSD) has been introduced in the ICD-11 and can be defined as the association of classic PTSD symptoms with severe and persistent disturbances of self-organization (DSO, namely, problems in affect regulation, beliefs about oneself as worthless, accompanied by feelings of shame, guilt or failure related to the traumatic event, and persistent difficulties in sustaining relationships and in feeling close to others (100). cPTSD affects around 1 to 8% of the general population and up to half of the patients in clinical population (100). Given that chronically traumatized patients are also at risk of developing borderline personality disorder, that cPTSD and BPD share clinical symptoms (notably the DSO symptoms), and given the important comorbid rates between the two disorders (101), there has been a large debate on the differences between the two disorders, with some authors proposing cPTSD as an alternative diagnosis for BPD patients (102) while other emphasizing the concurrent validity of the two diagnoses (103).

Recently, Ford & Courtois reviewed the existing literature on the link between PTSD, cPTSD and BPD and summarized important information regarding differences in comorbidity, neurobiology, and in several clinical dimensions including emotion dysregulation (23). Notably, they described emotion dysregulation in BPD as a difficulty in regulating intense distress related to real or perceived abandonment (the main trigger that we presented earlier), whereas emotion dysregulation would be better described as a difficulty in regulating trauma-related distress, primarily due to trauma-impacted beliefs about self and relationships in cPTSD. Moreover, regarding interpersonal style, authors describe cPTSD patients’ interpersonal style as a severe emotional detachment and tendency to avoidance in relationships, often marked by mistrust and feeling of worthlessness, while BPD interpersonal style is more characterized by intense fluctuations and tendency to interpersonal dependency (an aspect that we also described earlier) (23, 100).

#### 3.2.3 Autism spectrum stress disorder

Autism spectrum disorder (ASD) is a common and heritable neurodevelopmental disorder affecting around 1% of the general population that consists in impairments in social communication and interaction, hypersensoriality, stereotypic behaviors, sometimes associated with intellectual disability (104). Even though it is not a core symptom, emotion dysregulation has been found to be an important symptom in ASD patients (22, 105). Moreover, patients with ASD are also at risk for ED-related behaviors like self-harm or anger outbursts (21, 44). Regarding this issue, several authors has hypothesized that ASD patients may experiment ED in relation to core deficits of the disorder, such as difficulties in filtering environmental stimuli (including sensory) and cognitive rigidity (notably intolerance to change) (22, 105). Moreover, ASD patients’ interpersonal style is typically characterized by lack of understanding of social norms, lack of willingness to enter in relations with others that do not share their restricted interests, and with trouble understanding non-verbal communication and social reciprocity.

#### 3.2.4 Adult attention deficit hyperactive disorder

Attention-deficit hyperactivity disorder (ADHD) is a valid and recognized neurodevelopmental disorder affecting around 2.8% of the adult population (106) and is characterized by hyperactive-impulsive and/or inattentive symptoms with onset in childhood, before 12 years old (7). ED is considered as a central symptom of adult ADHD (25, 107), and several authors have emphasized its central role in externalizing behaviors and functional impairment in social and professional spheres (8, 25, 107). When looking at ED triggers in adult ADHD, we believe – as suggested by some authors (25) - that impatience and boredom are the most relevant one, especially in patients with hyperactivity, even though this aspect has not specifically been studied to our knowledge. Moreover, regarding interpersonal style, preliminary studies have found that ADHD patient often has difficulties caused by executive functioning difficulties, with troubles to keep contact with peers due to their impulsivity and novelty seeking, and with a global tendency to be perceived as too talkative and excitable (108).

## 4 Integration of current approach in diagnostic assessment

**Figure 1:**
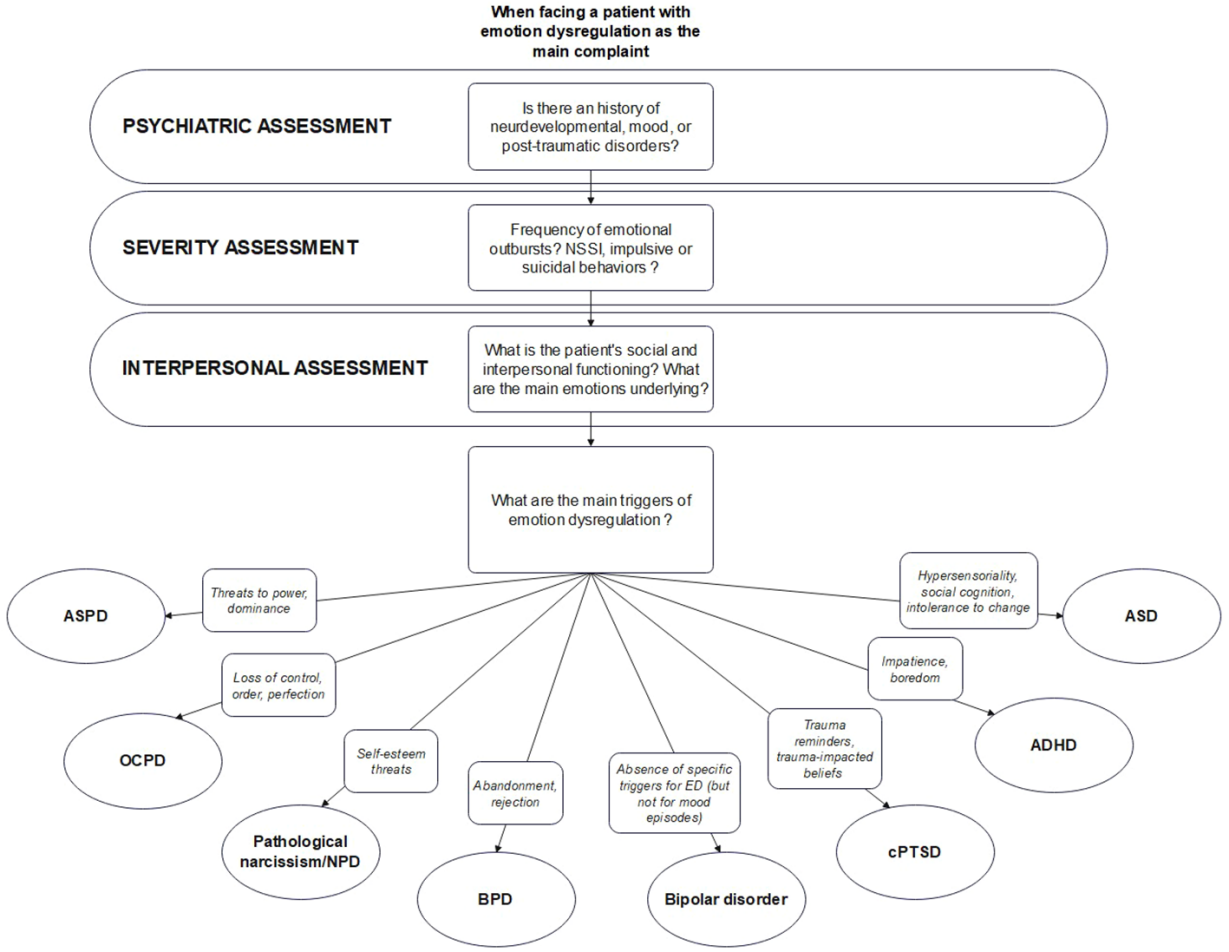
Sumary of our processual approach. ADHD, attention deficit hyperactivity disorder; ASD, autism spectrum disorder; BPD, borderline personality disorder; cPTSD, complex post-traumatic stress disorder; NPD, narcissistic personality disorder; OCPD, obsessive-compulsive personality disorder.

This model provides a simple clinical tool to help clinicians who are not familiar with the current literature on emotion dysregulation to better orientate their treatment by focusing more on the main difficulties rather than only on categories. In this paragraph, we will provide preliminary clinical diagnostic assessment guidelines for clinicians facing a patient with emotion dysregulation as the main complaint. A summary of these guidelines can be found in **Figure 1**.

When facing a patient whose chief complaint is emotion dysregulation, clinicians should start by systematically assess past/actual mood episodes (with an emphasis on the length of the episodes to avoid confusing mood and emotional swings), traumatic events (with or without post-traumatic stress syndrome), presence of autistic traits (including stereotypic behaviors and restricted interests), hyperactive or inattentive symptoms (appearing in childhood) or other neurodevelopmental issues (including learning disabilities) in their classic psychiatric examination. Then, clinicians should go further by assessing ED severity, notably through the investigation of the frequency of emotional outbursts and the presence/frequency of ED-related behaviors. Notably, clinicians should search for non-suicidal self-injuries [like cutting, biting, hitting, burning, scratching, but also less studied behaviors like high number of body modifications, especially tattoos and piercing, as they are linked to emotion dysregulation and are thought to be socially acceptable forms of NSSI by some authors (109)], impulsive behaviors in response to emotional outbursts (sexual endangerment, eating disorder, substance use, reckless driving and other types of orderly behaviors) and suicidal ideations/attempts. If present, each of these elements should be carefully explored in term of potential legal or physical consequences, notably somatic, especially for impulsive behaviors. This exhaustive behavioral assessment could be of great interest to estimate the potential severity and dangerousness of the syndrome. Then, when a clear behavioral picture is available, clinicians should continue by assessing social and interpersonal functioning, including patient’s perception and understanding of others, satisfaction and interest in social interactions, number of significant relationships, avoidance of social situations (with motives), and occurrence of conflicts (with motives). We believe that clinicians should put a large emphasis on the patients’ emotions typically associated with interpersonal interactions, like shame, anger, fear or guilt, and should try to understand how these emotions may be linked with ED-related behaviors. Finally, after this overall assessment, clinicians could start to focus on the underlying emotional triggers.

After a presentation of the main triggers listed in **Table 1**, and using all the information that has emerged throughout the interview (notably on psychiatric history and interpersonal style), clinicians could start a collaborative discussion with the patient and try to estimate the most important aspects that are triggering emotional crises. Once the main triggers are identified and linked with psychiatric history and interpersonal styles, clinicians should discuss with the patient how to hierarchize them, in order to target the most debilitating (i.e., that is the most commonly found in emotional crises) and to start to set treatment framework.

## 5 Trigger-based treatment orientation: general vs. specific interventions

Once specific triggers in terms of emotion dysregulation have been identified, we believe that clinicians should incorporate both general (i.e., applicable to every patient, regardless of the underlying triggers) *and* specific (i.e., linked to the underlying triggers) therapeutic interventions. In the next paragraph, we suggest several propositions regarding these interventions, again based on a narrative review of the literature. To make it more readable for readers, we decided to divide them in three main categories: psychoeducation, narrative work (i.e., building a person-centered understanding on how and why each difficulty has arisen), and goals setting. These interventions are summarized in **Table 2**.

**Table 2:** Summary of general and specific therapeutic interventions in terms of psychoeducation, narrative work, and goal setting.

Disorder		Psychoeducation	Narrative work	Goal setting
*Every disorder* *(General interventions)*		Focusing on the concept of ED, using DBT, MBT, or other evidence-based conceptualizations.	Focusing on the early signs of emotion dysregulation in childhood, and on the mismatch between the patients’ needs and what the environment provided.	Focusing on providing emotion regulation guidelines and learning chain analysis.
*Personality disorders*	**Borderline personality disorder**	Focusing on the link between ED and fear of abandonment and rejection.	Focusing on the early signs of intolerance to aloneness and rejection hypersensitivity.	Focusing on building a social role through employment and stable social tissue, rather than focusing on romantic relationships.
	**Narcissistic personality disorder/Pathological narcissism**	Focusing on destigmatization of the concept of pathological narcissism (notably through the use of self-esteem dysregulation models), and on the link between ED and self-esteem threats.	Focusing on how and why the grandiose self and self-esteem dysregulation developed (i.e., as an unconscious strategy to survive in an often highly criticizing and punishing environment).	Focusing on challenging grandiosity, notably through shame and vulnerability exposure, in order to build an integrated sense of identity relying on the patient’s true strengths and weaknesses rather than on an envisioned view of him/herself.
	**Obsessive-compulsive personality disorder**	Focusing on the link between ED and rigidity/perfectionism and on how obsessive-compulsive traits can be useful in some situations.	Focusing on how parental over-control, hyper-responsibility, and strong moral educative frame can lead to the development of obsessive-compulsive traits.	Focusing on challenging rigid and frightening beliefs and their consequences (procrastination, avoidance, perfectionism, self-criticism, hostility towards others) through corrective experiences and exposure, in order to develop an identity untied to rigid standards.
	**Antisocial personality disorder**	Focusing on the link between power and dominance threats and ED manifestations, and on the link between ASPD and comorbidities.	Focusing on how maltreatment, intrafamilial violence, child needs’ negligence, and a history of PD in the family may lead to the development of antisocial traits.	Focusing on building a strong treatment framework, responsabilizing the patient, identifying important values, developing mentalizing abilities, and regaining a social role through stable employment, while being attentive to contingency management and secondary gains.
*Other disorders*	**Bipolar disorder**	Focusing on depressive and manic episodes characteristics, on the differences between mood and emotion, and on the importance of medication.	Focusing on the early signs of the disorder (e.g., severe depressive episodes in adolescence) and on the link with family history.	Focusing on the equilibration, tolerance, and adherence to medication (notably through the development of a transparent therapeutic relationship), but also on the importance of overall life hygiene and self-monitoring of symptomatic relapses.
	**Complex post-traumatic stress disorder**	Focusing on the three dimensions of PSTD and their biological underpinnings, on the disturbance of self-organization symptoms, and on the construction of an individualized trauma-model (encompassing typical reviviscences triggers, maladaptive avoidance, and escape behaviors)	Focusing on *how and why* disturbance of self-organization symptoms appeared, with for example the use of the traumatic invalidation model.	Focusing on the access and conduction of specific trauma-focused psychotherapy (e.g., EMDR, TF-CBT, DBT-PTSD, MBT-PTSD), with possibly a special emphasis on motivation to change, skills-assisted exposure, and radical acceptance.
	**Autism spectrum disorder**	Focusing on social cognition, hyper-sensoriality, and intolerance to change issues, and on how these symptoms may be linked with ED.	Focusing on the notion of neurodevelopmental disorder and on the exploration of symptoms throughout patient’s history (e.g., childhood, adolescence, and adulthood).	Focusing on social cognition learning, with also an emphasis on the treatment framework (low-noise location, no unexpected changes in agenda) and on cognitive flexibility.
	**Attention deficit hyperactive disorder**	Focusing on hyperactivity and inattention symptoms, with a clear emphasis on their neurobiological underpinnings and on their link with ED. Also provide psychoeducation on medication.	Focusing on the notion of neurodevelopmental disorder and on the exploration of symptoms throughout patient’s history (e.g., childhood, adolescence, and adulthood), with also a clear emphasis on the link between ADHD symptoms and socio-educative issues.	Focusing on the importance of psychostimulant medication equilibration, tolerance, and adherence, but also of psychotherapy (notably through cognitive rehabilitation and development of compensating strategies).

In terms of general interventions, we believe that every patient suffering from emotion dysregulation should benefit from three main elements. First, psychoeducation on the concept of ED should be provided, with a focus on the concepts of emotional vulnerability and emotional invalidation (3), on mentalizing impairments (6), or on any other evidence-based conceptualizations of ED. Second, clinicians should highlight how and when such difficulties developed in their patients’ childhood and adolescence, with several examples, in order to build a coherent narrative of the apparition and perpetuation of ED. Third, general emotion regulation guidelines should be provided, the most commonly used being the DBT skills (developed by Linehan) and mentalizing training (developed by Bateman and Fonagy). Usually, such skills are taught in group format as parts of global psychotherapeutic programs (3, 110). However, these skills can also be useful outside their classic framework, an assumption underlined by their efficacy as a stand-alone treatment [e.g., for DBT (111)]. Thus, clinicians working with patients suffering from ED may implement several of these skills in their practice, either in session (if trained and possible) or through other means (e.g., online platforms, self-help books…). For example, some authors have suggested how YouTube videos made by professionals may be useful in this indication (112). Finally, another important general aspect of treatment for patients with ED is to provide basic guidelines on how to conduct what behavioral therapists call “chain analysis” (i.e., analyzing what leads to problematic behaviors). Indeed, the latter is central to gain awareness about the main processes at play in difficult situations and may be crucial to both the diagnostic and treatment processes. Once patients have identified the main elements leading to their ED manifestations, they may be able - with the help of their therapists - to try to change the typical pathological patterns.

Once these general elements have been provided, clinicians may start to work on more specific aspects, depending on what trigger(s) was(were) found in the diagnostic process. These specific interventions should be based on the core psychopathological processes and specific issues of each disorder. Thus, specific psychoeducation should focus on linking ED manifestations and specific triggers, specific narrative work should focus on how and why such triggers appeared throughout the patient history, and specific goal setting should focus on solving the underlying issues characteristic of the trigger.

Regarding personality pathology, for borderline patients, a specific emphasis should be made on intolerance of aloneness and fear of abandonment, and goal setting should be focused on building a life outside fusional relationships (i.e., employment, stable social tissue…) (45). For narcissistic patients, given the large amount of stigma associated with the construct (113), we believe that psychoeducation should focus on destigmatizing the concept (notably through the use of self-esteem dysregulation models) and should underline the core relevance of self-esteem threats, with goal setting focusing on challenging the envisioned view of oneself through shame and vulnerability exposure (75, 114). For obsessive-compulsive patients, psychoeducation should focus on explaining rigidity and perfectionism constructs, as well as the role of control and perfection threats in ED manifestations (79, 81). A discussion on the sometimes positive and adaptive aspects of these traits is also encouraged. On the other hand, narrative work should explain how parental over-control, hyper-responsibility, and strong moral educative frame can lead to the development of obsessive-compulsive traits, and goal setting should focus on living corrective experiences (through expositions to fearful and rigid beliefs) in order to build an integrated sense of self-worth (i.e., not relying only on effort and work) (79, 81). Finally, for antisocial patients, a specific emphasis should be made on building a strong treatment framework, with careful attention on contingency management and secondary gains, with psychoeducation on how antisocial traits typically develop (mistreatment, intrafamilial violence, child needs’ negligence…) and on the link between ASPD and comorbidities (mood, substance use disorders…) (115). Finally, specific interventions for ASPD patients should focus on identifying important values, developing mentalizing abilities (notably through group interventions), and regaining a social role through stable employment (116, 117).

Regarding other disorders, ADHD and bipolar patients care should include a specific attention on treatment equilibration, tolerance, and adherence (notably through the development of a transparent therapeutic relationship). For bipolar patients, it should also include psychoeducation on both types of episodes, with a narrative work on the early signs of the disorder (e.g., severe depressive episodes in adolescence) and on the link with a family history of bipolar disorder. Moreover, discussions should be conducted on the difference between mood episodes and emotions and on the importance of overall life hygiene and self-monitoring of symptomatic relapses (118, 119). For ADHD patients, psychoeducation should include a sensibilization to the concept of neurodevelopment, clear explanations on the symptoms of the disorder and their neurobiological underpinnings, and a discussion on the importance of cognitive rehabilitation and development of compensating strategies (120, 121). These patients should also benefit from a narrative work exploring ADHD symptoms throughout patient’s history (e.g., childhood, adolescence, and adulthood), to underline the link between their symptoms and socio-educative issues. For ASD patients, specific care should also include sensibilization on the concept of neurodevelopment, psychoeducation on ASD manifestations, with once again a narrative exploration of symptoms development throughout the patient’s history (44, 104). These patients should also benefit from social cognition and cognitive flexibility training, with a special emphasis on respecting hypersensoriality and intolerance to change. Finally, cPTSD patients should benefit from psychoeducation focusing on the PSTD triad and on the disturbance of self-organization symptoms, narrative work focusing on how and why DSO symptoms appeared [with for example the use of the traumatic invalidation model (122)], and goal setting focusing on the access and conduction of specific trauma-focused psychotherapy (e.g., EMDR, TF-CBT, DBT-PTSD, MBT-PTSD), with possibly a special emphasis on motivation to change, skills-assisted exposure, and radical acceptance (100, 122).

All these elements can be hard to implement concomitantly. Thus, clinicians should be aware of the risk of confusing patients through information overload (123). This underlines the importance of establishing a clear treatment hierarchy in terms of triggers, and a clear treatment framework in terms of interventions. Our main idea is not to offer *every* specific intervention to *every* patient suffering from one trigger, but more to adapt the treatment content individually for each patient.

## 6 Discussion

Given its transnosographic aspect, emotion dysregulation can be found in a large number of psychiatric disorders, and is considered by several authors to be central in the development, expression and maintenance of these disorders (8). However, in our current classifications, it is only emphasized in BPD criteria, and clinicians may be confused when facing a patient with emotion regulation issues, even more if this is associated with self-harm, impulsive behavior and suicide attempts, all often considered to be classic borderline symptoms. In this context, the probability of BPD co-diagnosis is high in those patients and may increase the likelihood of over-focusing on BPD relevant aspects (e.g., fear of abandonment and rejection) and thus neglecting other important aspects. We believe that a processual approach focusing on triggers (i.e., *what’s underlying* ED) and enhanced by an exploration of the typical interpersonal styles of the patient may be useful to improve diagnostic assessment and treatment orientation of patients suffering from ED.

Indeed, these high levels of comorbidity enlightens the flaws of our categorical approach, mostly because these multiples comorbidities do not orient towards the most relevant disorder for the patient. However, as mentioned earlier, categories are still useful, because they offer important opportunities, especially in terms of psychoeducation. We believe that focusing on the main psychopathological processes may help overcome this issue, by incorporating the positive aspects of categories (i.e., patient’s global understanding of his/her suffering) and setting aside its negative sides (e.g., rigidity and heterogeneity). Such approach could offer an interesting option for clinicians facing ED, especially in patients with multiple comorbidities, to help them determine, through a collaborative discussion with the patient, what aspect is the more invalidating and should be addressed first, and what aspect should be taken care of afterwards. This could allow to set goals that are closer to the patient’s experience and that does not only rely on BPD categorical diagnosis or behavioral aspects of emotion dysregulation, even though these are important aspects. This could also enhance the quality of therapeutic relationship. According to Fonagy & Allison (124), correspondence between the patient’s experience and what the patient thinks the therapist thinks he/she is experiencing is crucial in the establishment of a productive therapeutic relationship. By moving the focus from classic symptoms to recurrent interpersonal patterns, this approach could help the patient to become aware of the internal coherence of his/her behaviors, which may help to decrease self-stigmatization and contribute to generate epistemic trust towards the therapist. Finally, we believe that incorporating the patient at all steps of the diagnostic and goal setting process may continue to increase the patient’s trust and, thus, his/her active participation in the treatment, which would allow a better compliance in both its psychotherapeutic and pharmacological components.

One aspect that could be opposed to our approach is the fact that evidence based dimensional models incorporating emotion regulation issues already exists. We presented some of them in the introduction, and some might say that our model stands more as a duplicate than as a real contribution to the field, especially when considering the HiTOP model and its “emotion dysfunction” super-spectra. However, these models are mostly theoretical, and are only scarcely used in clinical practice. Clinicians are, for a vast majority, still using the categorical approach, with its flaws and limitations. Moreover, there is to date no specific treatment interventions based on these models, which is not the case for categorical approach. Thus, we are now in a complex situation where categorical approach is flawed but remains the most used and studied. In this context, we believe that our model offers an interesting in-between, offering the possibility to incorporate dimensional aspects, while remaining pragmatically based on classic categories.

With this model, our primary aim is to provide a simple transdiagnostic model for generalist clinicians who are not familiar with the current literature on dimensional approaches or ED, and that may need some help in their daily practice. Given the large increase in the scientific production in the last decades (125), clinicians often struggle to maintain up-to-date knowledge and to apply guidelines (126). As described earlier, we believe that this could lead them to overfocus on categorical diagnoses which may in turn lead to underestimation of relevant psychopathological processes that are not accounted in these categories. Providing simple and condensed knowledge is useful to overcome this issue (127, 128). In this paper, we tried to condense clinically useful information on ED in several disorders, while adopting a more didactic approach than classic systematic reviews, meta-analyses, or umbrella reviews. Simple models are easier to implement in daily practice, even though some might say that they are oversimplifying or incorrect. Our aim was not to conduct a systematic review of the current literature regarding ED (see (8) for an updated review), but more to participate in the current movement that highlights the importance of adopting a more dimensional approach to psychiatric disorders.

Finally, we also believe that a *trigger-based* approach could be useful not only in terms of diagnostic orientation, but also in terms of improving classic psychotherapeutic treatments. Several specific treatments have been developed to treat ED, the two most popular approaches being dialectical behavioral therapy [DBT (3)] and mentalization-based therapy [MBT (6)], initially developed for BPD patients. Adaptations of DBT and MBT have already been proposed for cPTSD (122, 129), ASD (44, 130), adult ADHD (131, 132), or ASPD (116, 133). Indeed, it seems legitimate to assume that, even if these patients present the same behavioral difficulties, given that the underlying core triggers are different, a part of the content of classic DBT and MBT programs should be modified to be more tailored to these different underlying difficulties. On a neurocognitive perspective, such adaptation could be understood as a way to target more specifically each disorder emotion regulation patterns, given that these triggers could be seen as the expression of different impairments in the emotion regulation cycle. Another example of the relevance of such adaptation can be found in another BPD treatment, Good Psychiatric Management, that has been adapted for NPD and OCPD patients. These adaptations emphasized the usefulness of a self-esteem centered approach for narcissistic patients (to take into account the NPD-specific therapeutic challenges) and a more control centered approach in obsessive-compulsive patients (with an emphasis on challenging the perfectionist and rigid standards of the patient) (75, 79). In this paper, we tried to summarize the main therapeutic contents that we believe are the most relevant for each disorder. One could assume that these propositions could be implemented in already existing specialized treatments for each diagnostic category. Even though this summary should not be taken as holistic and complete, we believe that it provides simple and preliminary guidelines that could (and should) be modified and improved in the future.

However, our model has several limits. First, it does not capture the complexity of each of the presented disorders, and it cannot be considered as definitive or holistic view of such disorders, or of emotion dysregulation. Second, it does not include every disorder associated with ED. We made a subjective selection based on our clinical experience in BPD-treatment centers, and further research is needed to support our clinical claims. Third, we chose to focus on only two aspects (triggers and interpersonal style), which may also be considered as subjective and incomplete. However, we believe that, in the emotion dysregulation construct and its related aspects, these two elements are the most likely to reflect underlying psychopathology, given that the other elements (like impulsivity, self-harm, suicide attempts) are almost purely behavioral. Fourth, there is currently a lack of comparative studies investigating the differences in terms of emotion regulation development, triggers and emotion regulation strategies between the 8 disorders. Thus, further research should be conducted on this aspect to confirm/infirm our theoretical claims. Fifth, this model mostly focuses on adult patients. Even though we tried to provide developmental perspectives on the origins of triggers that may also be relevant in adolescents, future theoretical and empirical works should be conducted on this specific population to potentially adapt some parts of our model to this population specificities. However, despite these important limitations, we consider that this model constitutes a useful tool for general (and maybe specialists) clinicians and may positively contributes to the development of a more dimensional and transdiagnostic approach of emotion dysregulation.

## 7 Conclusion

In this article, we presented a transdiagnostic processual model of emotion dysregulation based on core triggers and interpersonal styles. Our aim was to provide a simple but technical tool to help clinicians in the diagnosis assessment and treatment orientation of their emotionally dysregulated patients. By focusing more on typical patterns and interpersonal dynamics than only on categories, we believe that this model may contribute to the actual need for improvement of our current psychiatric classifications, alongside other well-studied and under-used dimensional models of psychopathology (e.g., HiTOP, AMPD…), and may be useful to build more specific treatment frameworks for patients suffering from ED.

## Data availability statement

The original contributions presented in the study are included in the article/supplementary material. Further inquiries can be directed to the corresponding author.

## Ethics statement

Ethical approval was not required for the study involving humans in accordance with the local legislation and institutional requirements. Written informed consent to participate in this study was not required from the participants or the participants’ legal guardians/next of kin in accordance with the national legislation and the institutional requirements.

## Author contributions

MB: Conceptualization, Writing – original draft, Writing – review & editing. MD: Conceptualization, Writing – review & editing. M-AD: Writing – review & editing. AD: Writing – review & editing. ER: Writing – review & editing. SW: Writing – review & editing. MS: Conceptualization, Supervision, Writing – review & editing. NP: Conceptualization, Supervision, Writing – review & editing.
